# Investigating mechanisms and monitoring outcomes in functional neurological disorder: the unrealized potential of digital remote monitoring technologies

**DOI:** 10.1098/rstb.2024.0471

**Published:** 2026-09-17

**Authors:** Susannah Pick, Matthew Hotopf

**Affiliations:** Department of Psychological Medicine, Institute of Psychiatry, Psychology & Neuroscience, King's College London, London, UK

**Keywords:** functional neurological disorder, psychogenic non-epileptic seizures, dissociative seizures, digital, wearable, mobile, outcome, mechanism, longitudinal, mHealth

## Abstract

Functional neurological disorder (FND) is characterized by involuntary, distressing neurological symptoms that are distinguishable from other neurological diagnoses in presentation, aetiology and mechanisms. Significant disability, impaired quality-of-life and substantial healthcare resource utilization often result from the disorder, yet its underlying mechanisms have not been proven empirically. Importantly, long-term clinical trajectories and predictive markers have been understudied in FND cohorts. Inadequate funding of large-scale mechanistic and longitudinal research, methodological limitations and the absence of standardized outcome measures necessitate development of innovative solutions. Digital remote monitoring technologies (dRMTs), which have been shown to be valuable across a range of other neurological and psychiatric disorders, may have substantial potential to address these research challenges in FND, yet this potential is currently unrealized. We propose that dRMTs could be harnessed to monitor subjective and/or objective functional neurological and associated symptoms, mechanistic variables and important outcome domains, with excellent temporal precision, flexibility and external validity. These methods could allow precise characterization of both short- and long-term fluctuations in FND symptoms, the influences on them and their impact on patients. Such data would strengthen mechanistic models, improve outcome prediction and facilitate personalized FND clinical management in this underserved population.

This article is part of the discussion meeting issue ‘Digital healthcare for the management of functional neurological disorders’.

## 1. Introduction

Functional neurological disorder (FND) is an umbrella diagnosis subsuming an array of sensory, seizure, motor and cognitive symptoms that can occur either in isolation or in conjunction [1,2]. Functional neurological symptoms (FNS) are involuntary, distressing and distinguishable from other neurological presentations in both aetiology and clinical presentation [3]. Distinctive clinical features include Hoover's sign in functional limb weakness, entrainment and distractibility in functional tremor, specific patterns of semiology in functional seizures, and internal inconsistency in functional sensory and cognitive symptoms [4–8].

The personal, clinical, and societal implications of FND should not be understated. FND is a common diagnosis, estimated to affect 50–100 000 people in the UK alone [9], and constituting up to approximately 15% of outpatient neurology consultations [10,11]. Patients with FND, and those close to them, describe a pervasive, life-changing impact of the disorder; it can diminish an individual's independence in daily activities, alter their sense of identity, life roles and goals, and impair their financial stability, occupational functioning and mental health status [12–15].

FND is associated with significant disability, impaired quality-of-life and substantial healthcare resource utilization, which can be similar, or more pronounced than in other major neurological diseases [16–20]. Nevertheless, there are significant and often impenetrable barriers to accessing appropriate treatment [21–26]. The disorder and its debilitating consequences too often persist in the long term [27,28].

Despite the clinical significance and widespread consequences of FND, there remains insufficient research funding allocated to the disorder and limited empirical evidence relating to its underlying mechanisms and clinical outcome trajectories. Unlike neurological disorders with established neuropathological causation (e.g. epilepsy, stroke, neurodegenerative disease), FND is conceptualized as a neuropsychiatric disorder arising from complex biopsychosocial underpinnings and multinetwork brain dysfunction [29–36]. However, there is limited evidence for the specific biopsychological mechanistic processes thought to be central to generating FNS [33,35,37] and patients report that incertitude and stigma relating to the aetiology and mechanisms of FND are a significant cause of distress [12–14,38–41]. Moreover, a lack of established outcome measures for FND leaves outcome monitoring inconsistent across interventional and cohort studies [42]. There is a paucity of large-scale, prospective, longitudinal outcome studies in the field, meaning that reliable predictors of prognosis and treatment response are yet to be established [27].

Here, we propose that digital remote monitoring technologies (dRMTs) have considerable, underexploited potential for addressing some of the challenges in FND research, focusing specifically on mechanistic research and outcome monitoring.^1^ We summarize how dRMTs have been applied in other neurological and psychiatric populations, before presenting a rationale for using dRMTs in research on FND mechanisms and outcomes, and outlining the small number of existing studies that have used dRMTs for these purposes to date. Finally, recommendations are presented for future research in this space.

## 2. Digital remote monitoring technologies: optimal tools for real-world neurological and psychiatric research

Digital RMTs are electronic devices with data acquisition, storage and computational capabilities, such as wearable and portable devices (e.g. watches, bracelets, smartphones). *Passive dRMTs* include inbuilt sensors in wearable or other devices that can unobtrusively monitor a variety of patient-related and environmental variables. For example, certain symptom types, particularly those involving abnormalities of movement, can be monitored directly with passive dRMTs, such as accelerometery-based measurement. Passive dRMTs can also acquire signals that offer insights into a wide range of other central nervous system functions, including physical activity, geolocation (providing information on daily routines), sleep and circadian rhythms, stress (e.g. heart rate variability), sociability (e.g. Bluetooth handshakes, phone-call/messaging frequency) and speech parameters. These domains are either difficult to measure objectively in real-world settings or require intense and costly in-person assessments, which may lack ecological validity.

For example, traditional polysomnography may provide ‘gold standard’ measures of sleep architecture, but it requires expensive technology in a sleep laboratory with relatively invasive electrophysiological monitoring. By contrast, sleep measured using a wearable device, detecting little more than movement and heart rate, can provide continuous, non-invasive and ecologically valid input over many months or even years. The two technologies provide different information, and while purists might be concerned at the validity of models used to provide information on sleep architecture from wrist-worn wearables, the data provided are simply different, for example, by monitoring the effects of changes in sleep over time on health, something which is not feasible in classic polysomnography.

In neurology, wearable sensors have been deployed to monitor the occurrence, features and/or severity of primary neurological symptoms across a range of diagnoses, including Huntington's disease, Parkinson's disease, epilepsy, multiple sclerosis and stroke [43–49]. Sleep, physical activity and mobility-related functional outcomes have also been monitored with accelerometery and GPS in patients with various neurological disorders [46,50–55].

In psychiatry, wearables have been used to obtain data passively on physiological and functional variables such as autonomic activity, sleep, physical activity, social interactions, speech parameters, and others [56]. In depression, for example, a growing number of small studies have reported results mainly using case-control designs to compare people with depression to healthy controls using passive dRMT methods [57]. The RADAR-CNS consortium has explored a multitude of RMT parameters related to depressive symptoms, demonstrating that people reporting more depressive symptoms tend to have less regular sleep, stay at home more and speak more slowly, at lower volume [58–61]. Such cross-sectional associations, while showing face validity of the RMT measures, do not necessarily tell us more than a patient or clinician would have known already. More recently, RADAR-CNS has attempted to investigate the fluctuation of symptoms within individuals and make predictions. One example is in sleep, where depressive relapse was predicted, with a population attributable fraction of 22%, by changes in sleep parameters the previous month [62]. Similar results have been found in schizophrenia with passive dRMTs [63]. Such passive dRMT measures could lead to prevention or early detection of relapse in these disorders, possibly with low cost and scalable interventions targeted at variables such as sleep or social functioning.

*Active dRMTs* require patients to voluntarily provide data using digital devices within their regular daily routines and natural environments. Active dRMTs utilize smartphone applications to acquire fine-grained information pertaining to subjective symptoms, other health-relevant daily experiences (e.g. activities, environmental events, subjective stress), and cognitive, affective or physical functioning. For example, administration of traditional, self-report measures can be made more convenient and accessible using online or smartphone-based survey platforms (e.g. Qualtrics, REDCap), allowing remote delivery of validated clinical scales to assess symptoms retrospectively over periods ranging from 1 week to several months (e.g. [64]).

More intensive questionnaire-based methods include ecological momentary assessment (EMA, or ‘experience sampling’) [65]. EMA involves administration of items assessing short-term experiences in the moment or in the very recent past (minutes/hours), with data entry prompted by notifications multiple times daily, or with data entry initiated by the participant when specific events occur. Methods like EMA circumvent the problem of retrospective reporting biases that are inherent in traditional clinical scales. Importantly, EMA facilitates tracking of moment-to-moment and day-to-day variability in subjective symptoms and other relevant experiences, thereby providing superior temporal resolution and accuracy, as well as providing critical insights into predictive temporal relationships between aetiological and mechanistic variables and symptom occurrence or aggravation [66]. The value of EMA-based methods in psychiatric research is well established for investigation of the phenomenology of psychopathological symptoms, their underlying mechanisms and in monitoring outcomes and/or predicting onset or recurrence of clinical episodes (e.g. [66–70]).

Digital cognitive testing is another key application of active dRMTs. While traditional neuropsychological testing of cognition provides gold-standard measurement accuracy, each assessment may take several hours of experienced neuropsychologist time, often requiring in-person clinic visits, resulting in high cost and time investment for both the clinical service and the patient, as well as limiting external validity [71]. Diverse cognitive tests are now available for digital administration (e.g. [71–76]), which allow low-cost, high-throughput testing on a much greater scale, with improved convenience and accessibility for patients, albeit with less established psychometric properties and quality [77]. Automated batteries can be administered using smartphones, tablets or web-based platforms, allowing cognitive assessments to be conducted online, remotely and repeatedly from patients’ own homes. As such, it is possible to ascertain strong baselines and to intensively monitor changes in cognitive functions over time in relevant clinical and pre-clinical populations [78–82].

In summary, dRMTs offer considerable opportunities to track core symptoms, other health-related domains, and potentially to predict outcomes or stratify patients with diverse neurological or mental health conditions. Such methods can track short- and long-term variability in primary symptoms and associated clinical features, with stronger temporal fidelity and external generalizability than traditional methods. These tools have potential to yield data on which to build powerful clinical prediction models, and may facilitate development of novel, targeted treatments. Furthermore, they have potential to assess the real-world impact of symptoms and to be accessible to those who may reside in remote locations, or who face other barriers to attending specialist clinical settings, such as marked physical disabilities, severe mental health symptoms or financial constraints.

Below, we discuss two major research challenges in the FND field that would benefit from improved implementation of dRMT methods: investigation of mechanisms and monitoring of symptoms and other outcomes.

## 3. Progress and unresolved issues in functional neurological disorder mechanistic research

Contemporary perspectives on the aetiology of FND acknowledge that the functional brain disturbances thought to underlie FNS develop within the context of complex and variable biopsychosocial aetiological influences [29,37,83,84]. Findings based on clinical data, qualitative methods or retrospective self-report measures suggest that a combination of biopsychosocial factors might predispose to and/or precipitate the onset of FND, including exposure to adverse life events and other chronic, inescapable or complex stressors, physical and/or mental health disorders/crises, physical injuries or accidents, and major medical procedures [14,31,32,34,36,84–88]. Maintaining factors may include suboptimal cognitive and behavioural responses to symptoms, social isolation or other relationship disturbances, and iatrogenic harm resulting from inappropriate medical interventions, stigmatization or discriminatory treatment from healthcare professionals [14,39,88–91].

Despite developments in biopsychosocial aetiological frameworks for FND, the pathways by which these influences culminate in the emergence of FND are unclear. Furthermore, the proximate causes driving short-term initiation or exacerbation of FNS have not been established definitively. Explanatory mechanistic models differentially emphasize disruptions in agency perception, salience processing, sensorimotor functioning, interoception, emotional awareness/reactivity, dissociation and predictive processing [29,31,35,37,92,93]. However, there is limited direct empirical evidence for these presumed causal influences on FNS occurrence or exacerbation, and there are methodological concerns relevant to many studies in this field [33,35,37].

For causality to be inferred, several criteria should be met, including (but not limited to) the following: (i) the outcome (e.g. FNS occurrence/exacerbation) and hypothesized causal variable must be strongly correlated, (ii) the change in the outcome should be temporally preceded by the change in the hypothesized cause, (iii) there must be a plausible mechanism underlying the influence of the cause on the outcome, and (iv) other probable explanations for the change in the outcome should be eliminated [94]. Few studies on FND mechanisms to date meet these criteria, in part owing to omission of subjective or objective FNS measurement in temporal contiguity with the hypothesized mechanistic variables [95,96].

Crucially, much of the available mechanistic evidence in FND is based on data obtained within artificial environments at single timepoints, or a limited number of timepoints, such as clinical settings (e.g. neuropsychological testing, diagnostic interviews, video-encephalography outcomes), neuroimaging facilities (e.g. magnetic resonance imaging data) and laboratories (e.g. behavioural experimental studies), which restricts the external generalizability of the findings. There is less known about the short-term antecedents of FNS in real-world naturalistic settings, which could yield data that would illuminate underlying causal mechanisms as they occur in daily life. Short-term FNS triggers retrospectively reported by patients vary considerably across cases, but commonly include acute stress, autonomic arousal or specific emotional states, trauma reminders, physical activity, cognitive exertion, fatigue, pain and sensory stimulation [86,90,97–101]. While valuable in individual cases, such retrospective reports may be distorted by attentional or memory biases and therefore offer limited information at the FND population level.

To provide convincing evidence for causal influences on FNS occurrence, it is necessary to establish valid and reliable means of measuring the core symptoms and their presumed causes, in ways that can be applied across clinical settings, laboratory-based and neuroimaging studies, and essentially, in real-world environments. FNS measurement tools for mechanistic research need to have high temporal resolution, so that we can track short-term changes in symptoms concurrently with hypothesized causal and mechanistic variables, to assess their temporal relationships and rigorously test causal hypotheses. Digital RMTs may be one important part of the solution to this problem, having the capability to intensively sample both subjective and objective variables, including core FNS, across situational contexts, with temporal precision and minimal burden on patients.

## 4. Functional neurological disorder outcome monitoring: debates and emerging consensus

Previous work from the FND Core Outcome Measures (FND-COM) group highlighted the challenges involved in accurately monitoring core symptoms and other outcome domains in patients with FND [13,42,102]. FND can present with a wide range of primary symptoms, which can vary substantially between individual cases and within cases over time, such as when new FNS emerge following the initial complaint [2]. Therefore, identifying which FNS should be tracked as a primary outcome can be problematic.

The frequency, severity and impact of FNS fluctuate strikingly from day-to-day and, in some cases, from hour-to-hour, meaning that single ‘snapshot’ clinical assessments of symptoms can be unrepresentative of the true symptom burden experienced. Similarly, retrospective self-report measures of symptom severity or frequency, which cut across time periods of weeks or months, can be biased by recall, current mood state or physical symptoms experienced, and may fail to provide adequate granularity regarding the peaks and troughs that took place during that interval.

Subjective and objective measures of FNS can often diverge [103–105], meaning that attempts to quantify symptoms using traditional, objective assessments, such as performance tests or standardized clinical investigations, may not adequately reflect the patient's ongoing symptom experiences. Complicating matters further, many patients with FND experience a range of comorbid physical and mental health symptoms, including pain, fatigue, depression, dissociation and anxiety, resulting in considerable additional impact and distress, and which can be closely associated with the intensity of the core FNS [86,106–111]. Which of these symptom types should be considered core to FND and/or included in FND outcome measures is a matter of debate [42,102].

As a result of these challenges, among others, there is currently no single, widely endorsed and validated outcome measure for FND [42,102]. However, consensus opinion concluded that subjective, patient-reported measures should be prioritized over objective ones. It was agreed that several additional outcome domains should be evaluated routinely with established, generic tools, covering primary symptoms, mental health, broader physical symptom burden, health-related quality-of-life, general functioning, disability and healthcare resource use [42]. Furthermore, the FND-COM group outlined the possibility of adopting a personalized approach in which patients select their most impactful symptom and report on this throughout the monitoring period, as well as highlighting the potential value of exploring discrepancies between objective and subjective outcome measures as a means of gaining insights into symptom or therapeutic mechanisms [42].

Owing to barriers facing patients in accessing diagnosis and clinical management, as well as research funding limitations, long term, prospective outcome monitoring in large FND samples has been limited, with few longitudinal prospective cohort studies completed [27,112–114]. Consequently, clinical trajectories and treatment response profiles in FND are less well detailed than other clinical syndromes with similarly disabling symptoms, such as epilepsy, Parkinson's disease and multiple sclerosis. Identification of robust biopsychosocial predictors of long-term outcome trajectories and treatment response profiles in FND would enhance an individualized, targeted approach to clinical management in this group.

There is a clear need for further development of acceptable, cost-effective, reliable, valid, scalable and protractible FND outcome monitoring solutions. Incorporating dRMTs into FND outcome measurement initiatives may thus have significant advantages. Digital RMTs are well suited to monitoring outcomes in FND, owing to their capability for simultaneous monitoring of both subjective experiences and objective markers. Feedback to patients from dRMT measures during or following an intervention could improve insight, understanding and appraisal of their symptoms, which might facilitate engagement with treatment and improve outcomes. The strong temporal resolution and flexibility that can be achieved with dRMTs would enable characterization of patterns of fluctuation in a variety of different FNS, including detection of long-term trends towards improvement or deterioration. These methods could pave the way towards time-critical preventative or interventional measures in those patients at most risk of the occurrence or worsening of clinically significant symptoms. Importantly, in our recent qualitative study [13], patients, carers and experienced FND healthcare professionals expressed the perceived value and acceptability of using dRMTs in outcome measurement in FND, suggesting that these methods might be well received in FND patient and clinician populations.

## 5. Digital remote monitoring technologies for investigating mechanisms and monitoring symptoms in functional neurological disorder: preliminary findings

Despite the methodological strengths of dRMTs, their wide use in related clinical populations and their apparent value to important stakeholders, there have been only a small number of studies using dRMTs to investigate mechanisms or monitor outcomes in FND to date.

Several examples of dRMT in FND research focused on monitoring functional motor symptoms, particularly functional tremor. The first examined the frequency of subjective and objective functional tremor with daily paper-and-pen symptom diaries and wrist-worn actigraphy spanning a 5-day interval [104]. Key findings were that the functional tremor sample (*n* = 10) reported significantly greater tremor duration than an organic tremor control group (*n* = 8), whereas the converse pattern was observed for actigraphy-based tremor frequency (objective tremor greater in the organic group). Additionally, greater concordance was demonstrated between the subjective and objective tremor measures in the organic sample compared to the functional group, and the latter group displayed tremor frequency in the normal range. Despite the absence of objectively verifiable tremor in the functional group, they reported significantly greater disability and poorer quality-of-life than the organic sample. This study was novel in its approach and influential in its findings, providing a clear demonstration of how subjective perceptions of symptoms can differ from objective markers in FND. While the sample size was small and the monitoring period short in this study, Dominguez-Vega *et al.* [115] subsequently provided evidence for the reliability of wrist-worn accelerometery in functional (*n* = 16) and organic (*n* = 23) tremor patients, concluding that 3 days of 6-hour monitoring was sufficient for extraction of reliable data.

Another study replicated some of these initial findings in a larger sample (functional tremor = 14; organic tremor = 19) over a period of 30 days, with digital online symptom diaries completed five times daily, alongside wrist-worn accelerometery [116]. Resembling the previous findings, objective tremor duration was reduced in the functional group relative to their organic counterparts; however, distinct results were that: (i) objective tremor was objectively demonstrated for some of the time in the functional group, (ii) subjective tremor was comparable between the samples reported, and (iii) the degree of association between subjective and objective tremor measures did not vary between the groups [116]. Nevertheless, the FND group reported greater overall symptom severity on the Clinical Global Impression scale, as well as greater difficulties with daily activities, compared to the organic group. This study provided overall corroboration of the impression of disproportionately elevated subjective symptoms and/or impairment in functional tremor patients, which were discordant with symptom severity ascertained with objective methods.

Kramer *et al.* [117] reported further data from this study on possible temporal relationships between subjective and objective functional tremor and negative affect, representing the first attempt to objectively explore the potential mechanistic role of emotion on FNS using dRMTs in a naturalistic context. Both time-lagged and concurrent analyses were performed to assess predictive relationships between tremor and negative affect across timepoints, in addition to associations between these variables at the same timepoint. The authors reported limited predictive relationships between affect and tremor in both FND and neurological controls, showing variability in direction and magnitude at the individual participant level in both groups. Modest concurrent associations between tremor and affect were observed in the functional and organic groups, which were weaker in the former group, and causal relationships could not be inferred. Together, these findings did not provide evidence for a specific, directional influence of negative affect on functional tremor, but the study demonstrated the utility of dRMT for testing hypotheses about mechanistic variables in real-world settings, laying a foundation for future work.

Another research group used accelerometery to objectively assess the prevalence of restless leg syndrome in participants’ home environments over three successive nights. This case-control study included 96 patients with mixed functional movement disorders and 76 healthy controls [118], who all underwent a specialist clinical consultation at baseline to assess the presence of restless leg syndrome, based on applying established diagnostic criteria to patients’ subjective symptom reports. Rates of restless leg syndrome were found to be higher in the FND group compared to controls, on both clinical and accelerometery measures. This study supported the potential usefulness of using dRMT to measure additional physical symptoms with accelerometery in FND samples, beyond the primary FNS.

Only two studies used dRMTs to monitor symptoms and examine possible antecedents in patients with functional seizures, beyond those used for diagnostic and seizure classification purposes (e.g. [47]). Mousa *et al.* [119] conducted the first study using actigraphy to inspect objective sleep quality in patients with functional seizures (*n* = 17) compared to healthy controls (*n* = 20) over 6 nights, combined with daily paper-and-pen diaries to monitor subjective sleep quality, seizure occurrence, affect and dissociation. Both objective and subjective sleep quality were diminished in the functional seizures group relative to healthy controls. Yet, within the functional seizures group, superior sleep quality (operationalized as fewer awakenings and longer duration) predicted a higher probability of seizure occurrence in the subsequent day, counter to the authors’ predictions. These results highlight the value of dRMTs for probing objective relationships between potential aggravating factors and symptom occurrence in this group, revealing a mismatch between what patients subjectively report and objective data.

In our recent pilot dRMT study [110], we asked participants with functional seizures or motor symptoms (*n* = 17) to select and track their primary (most impactful) FNS using both notification-initiated (eight times daily) and user-initiated (24/7) EMA over a sampling period of 7 days. Our participants also reported on subjectively significant daily events (i.e. uplifts, hassles, stressors), positive and negative affect, dissociation and other common physical symptoms (pain, fatigue, sleep disturbance). We combined these subjective measures with a wearable device (Fitbit Charge 5) to test several primary mechanistic hypotheses, focusing on the potential influence of autonomic arousal (heart rate, electrodermal activity), negative affect and stressful daily events on the occurrence or severity of FNS [33]. An age- and sex-matched healthy control sample completed the same protocol (without reporting on FNS). Even with this modest sample, across the week-long sampling period, we observed statistically notable differences between groups on several variables, including significantly elevated negative affect, dissociation, pain, fatigue, subjectively significant/stressful events, resting heart rate and sleep disturbance in the FND group. Time-lagged within-day analyses showed that the strongest temporal predictors of FNS occurrence or exacerbation from one timepoint to the next were subjectively significant daily events and heightened negative affect. Other relevant predictors in the time-lagged momentary analyses were pain, fatigue and (lower) positive affect. These findings converge with other results from this larger project, including symptom triggers reported by patients [86] and laboratory-based experimental testing [96].

We also monitored contextual variables within our EMA protocol at each prompt, including self-reported location, social context and activities engaged in immediately prior to the notification [110]. Our results showed a number of potentially important differences between the FND and control groups, including a greater likelihood in the FND group of being at home, with family or pets, and engaged in rest or passive leisure activities, relative to controls, the latter being more probably to be in public or work environments, with friends or alone, and engaged in more active pursuits. These results demonstrate the potential impact of FND in terms of homestay and restricted activities, suggesting the probable value of monitoring broader real-world outcomes with dRMT, beyond core FNS, such as social functioning, quality-of-life and disability. Measurement of such outcomes could be valuable in long-term prognostic research and in interventional trials.

To our knowledge, only one study has used dRMTs to monitor outcomes in an interventional study to date. In a feasibility randomized controlled trial of eye movement desensitization and reprocessing, Cope *et al.* [120] used a daily EMA protocol to monitor primary FNS for periods of two weeks at baseline and at each follow-up point (three, six and nine months), using a smartphone app to report on the frequency, severity and impact of the symptoms. Outcome data from the EMA measures have not yet been published, but completion rates were promising at baseline and earlier follow-ups.

## 6. Challenges and limitations in digital remote monitoring technologies research

One significant challenge in dRMT research is that the methods require active engagement and commitment from participants over prolonged periods of time, often spanning weeks or months. In clinical disorders that are associated with functional impairment, disability and/or waxing and waning motor, cognitive and psychological symptoms, engagement with dRMTs can be affected, potentially resulting in substantial missing data. User- or device-related technical issues with digital instruments can also disrupt collection of continuous, high-quality data, requiring strong technical and user support for troubleshooting throughout projects.

Despite the challenges noted, we observed a completion rate of 80% using our intensive EMA protocol alongside a wearable device in our pilot work [95,96], which we have recently replicated in a larger scale study. Completion rates in the range of 80% or more have similarly been shown in other FND dRMT studies [104,115–117]. Moreover, RADAR-MDD demonstrated high rates of dRMT engagement and retention in patients with major depression, with 80% of the cohort participating for the full length of the study [64]. Together, these completion rates are encouraging, suggesting that with a strong design, including patient and other stakeholder input, dRMT studies are feasible and acceptable in FND and related populations. However, many of these studies were short term and included incentives for engagement with the dRMTs, such as monetary reimbursement or provision of a device for personal use; therefore, it is unclear whether the same level of engagement would occur in longer term dRMT studies with less direct reinforcement.

Qualitative data have highlighted several facilitating factors and barriers to engagement and adherence with dRMT studies in patients with FND and related neuropsychiatric populations [110,121,122]. Perceived facilitators were strong support and communication from research or clinical teams, adequate, clear training and instructions, user-friendliness, flexibility and acceptability of the devices and protocols, perceived relevance of measures included (i.e. aiding understanding, prediction or self-management of symptoms), incentives for study completion, data security and accuracy of the devices. Nevertheless, perceived barriers included periods of worsened clinical status, potential for increased stigma and symptom-focused attention or distress, technical challenges (e.g. use of device, internet connectivity), as well as difficulties with engagement owing to other personal or professional commitments.

There are important ethical issues to consider in dRMT research. One issue pertains to the potential for negative consequences of intensive symptom monitoring in patients with FND, who could experience worsening of FNS and/or distress by having their attention repeatedly directed towards their symptom experiences (e.g. EMA). A randomized controlled trial could provide useful evidence regarding the possible benefits or detrimental consequences of dRMTs on FNS. In the meantime, participants in dRMT studies should be reminded routinely that they can desist from the research protocol at any time if they experience adverse effects. Studies should also be designed to be as accessible as possible to individuals from all sociodemographic groups, including those who may not have personal access to digital devices owing to financial constraints, or who may experience difficulties using digital technologies due to age, unfamiliarity, disability, language or other factors. It is vital to ensure the security and confidentiality of data collected via dRMTs, particularly when third-party data hosting services are involved. Participants should receive detailed information about how their data will be collected, stored and used, prior to consenting. Opt-out choices can also be made available for more sensitive aspects of monitoring (e.g. geolocation). It is essential that the digital data are fully anonymized to any third parties, and that participants are explicitly instructed not to provide any personally identifiable data using the devices.

While the preliminary findings outlined here illustrate the potential that dRMTs hold for mechanistic and outcomes research in FND and related disorders, there are several limitations in the existing literature as follows:

- Small sample sizes (<20) predominate in FND dRMT studies.
- A limited number of studies used intensive daily subjective assessments (EMA) using multiple, randomized or pseudorandomized sampling each day.
- EMA measures are largely unvalidated in FND and related populations.
- Missing data and confounding variables can be difficult to explain and/or statistically correct for, and may be correlated (e.g. the most severely unwell patients may be most likely to miss notifications and/or drop out of dRMT studies).
- There are no long-term dRMT studies, with sampling durations limited to a maximum of one month, which may not adequately represent longer term fluctuations in symptoms (i.e. remission and relapse).
- Some studies failed to adequately characterize samples at baseline, resulting in missed opportunities to examine convergence between traditional and dRMT phenotyping.
- Few studies have adopted research-grade wearables to objectively monitor variables other than accelerometery.
- Subjective FNS reports may be influenced by expectations, beliefs and suggestions.
- Few studies have combined objective measures (e.g. physical activity, sleep, autonomic variables) with *momentary* subjective reports of symptoms or other relevant variables.
- Patient and other stakeholder input has not reliably been sought in study design or implementation.

## 7. Research priorities for using digital remote monitoring technologies in future functional neurological disorder mechanistic and outcomes research

We propose several essential priorities for future research using dRMTs to investigate mechanisms and monitor symptoms in FND, as follows:

1. *Patient, carer and other stakeholder input* should be sought at all stages, including the planning, piloting, implementation, interpretation and dissemination of dRMT research in this population, to ensure acceptability, feasibility and relevance, and to maximize engagement.
2. *Ethical issues* should be considered in detail during the design and conduct of dRMT studies, including monitoring and mitigation of any potential adverse effects of dRMTs, enabling accessibility to under-represented sociodemographic groups and providing participants with detailed information about how their data will be managed, with opt-out options for each digital outcome variable.
3. dRMTs should be used to *track the occurrence, variability and impact of subjective FNS* in patients’ daily lives, using digital symptom monitoring protocols such as notification- or user-initiated EMA. These methods can be adapted to capture the full range of FNS types, but validation work is needed to establish the psychometric properties of such measures before they can be widely adopted in funded studies or routine practice.
4. dRMTs can be a means of *objective FNS monitoring* (e.g. accelerometery, objective cognitive testing), with more evidence needed for the reliability and validity of these methods in specific FNS subtypes (e.g. functional motor/seizure/cognitive symptoms), and use of research-grade wearables and sensors, where appropriate.
5. *Synchronous objective and subjective FNS measurement* with dRMTs should be used to highlight points of convergence and divergence between symptom perceptions and objective occurrence of FNS, in real time, providing theoretically and clinically relevant insights.
6. Monitoring *contextual variables* (e.g. location, social context, activities) with methods such as EMA could provide important data on functional outcomes such as occupational and social functioning, mobility and activities of daily living, which might complement the use of traditional patient-reported outcome measures.
7. *Measurement of biopsychosocial causal/mechanistic variables* within dRMT protocols will provide meaningful data on short- and long-term influences on FNS occurrence, including autonomic activation, psychological states, social and environmental variables, and other physical factors (i.e. sleep disturbance, fatigue, activity).
8. *Long-term outcome studies using dRMTs with large samples* (e.g. duration > 1 year, *n* > 100) would provide stronger data on clinical outcomes in FND, with greater statistical power, including use as outcome measures in interventional trials.
9. Combining *deep phenotyping* (e.g. clinical, lab-based, neuroimaging) with *longitudinal monitoring* with dRMTs will allow the development of robust and clinically relevant prediction models based on identifiable clinical, biological and psychological markers.
10. Examining the *convergence of dRMT data with clinical, lab-based and neuroimaging findings* will provide stronger empirical support for causal influences on FNS, to provide the foundation for more robust evidence-based mechanistic models.
11. Studies using dRMTs for mechanistic research should include relevant *control groups*, such as patients with other neurological diagnoses, or those with relevant psychiatric symptoms (e.g. depression, anxiety, trauma-related or dissociative symptoms), to assess the specificity of findings to FND.
12. *Open science practices* should be adopted in future dRMT studies, with pre-registration of protocols and detailed analytic plans, as well as sharing of anonymized data, data dictionaries and other relevant details, to ensure transparency and reproducibility, and to allow data synthesis in meta-analyses.

Our recent large-scale dRMT study [95] sought to achieve some of these aims in a sample of patients with FND, including individuals with functional seizures (*n* = 50) or functional motor symptoms (*n* = 50), compared to healthy (*n* = 50) and clinical (psychiatric) controls (*n* = 50). At baseline, participants underwent intensive in-person phenotyping assessments, undertaking in-depth clinical interviews, validated self-report measures covering biopsychosocial aetiological factors, laboratory-based assessments of cognitive, interoceptive and autonomic functioning, in addition to structural and functional neuroimaging sessions. We adapted our pilot dRMT protocol [110] for use in this longer term study, which included a six times daily notification-initiated EMA schedule across 14 days, with user-initiated event and symptom logging over four weeks, combined with continuous Fitbit Charge 5 monitoring of relevant physiological signals (sleep, heart rate, physical activity). Participants were invited to continue wearing the Fitbit devices and provide data for up to 1 year, in addition to completing online self-report clinical measures and a brief follow-up interview at three, six and 12 months following baseline phenotyping. In our total sample of 200 participants, 187 consented to the 14-day dRMT phase and 185 completed it, with an average EMA response rate of 82% across the groups (80% in the FND sample, *n* = 100), and 183 participants provided Fitbit data. These preliminary data indicate excellent engagement with the intensive dRMT element of the project, supporting our earlier findings that these methods are acceptable, feasible and relevant to patients with FND.

## 8. Conclusions

A comprehensive, evidence-based explanatory model of the causal mechanisms underlying FNS is yet to be established definitively. Furthermore, challenges impeding accurate and reliable monitoring of FNS and other outcome domains result in insufficient knowledge regarding long-term clinical outcomes in FND and the markers that predict them.

Digital RMTs have been shown to be valuable tools in mechanistic research and outcome monitoring/prediction in a range of other psychiatric and neurological disorders; however, this potential is yet to be realized in FND. Previous studies using dRMTs for investigation of FND mechanisms and/or outcome monitoring have been limited in scale, duration and/or study design.

We propose that dRMTs offer myriad opportunities to: (i) generate externally valid, large-scale data on causes and mechanisms underlying FNS occurrence; (ii) optimize short- and long-term outcome monitoring; and (iii) develop robust and precise predictive models that could aid personalized management approaches in FND. Insights derived from dRMT research, in combination with other modalities (e.g. clinical, neuroimaging, laboratory-based testing), will improve the evidence base and coherence of explanatory models, indicate which patients should be prioritized for specific treatments, and may yield information that can enhance self-awareness and management of symptom triggers and aggravating factors in everyday life.

## Data Availability

This article has no additional data.

## Footnote

^1^
Use of dRMTs for purely diagnostic or interventional purposes is not considered in this manuscript.

## Theme

One contribution of 12 to a discussion meeting issue ‘Digital healthcare for the management of functional neurological disorders'.
